# Comparison of Blood Pressure Variability between 24 h Ambulatory Monitoring and Office Blood Pressure in Diabetics and Nondiabetic Patients: A Cross-Sectional Study

**DOI:** 10.1155/2022/1022044

**Published:** 2022-06-21

**Authors:** Ana Lídia Rouxinol-Dias, Marta Lisandra Gonçalves, Diogo Ramalho, Jose Silva, Loide Barbosa, Jorge Polónia

**Affiliations:** ^1^Center for Research in Health Technologies and Information Systems (CINTESIS), Faculty of Medicine, University of Porto, Porto, Portugal; ^2^Department of Anesthesiology, Centro Hospitalar Universitário de São João EPE, Porto, Portugal; ^3^Department of Medicine, Faculty of Medicine, University of Porto, Porto, Portugal; ^4^Endocrinology Department, Centro Hospitalar Vila Nova de Gaia e Espinho EPE, Vila Nova de Gaia, Portugal; ^5^Hypertension Unit/ULS Matosinhos EPE, Matosinhos, Portugal

## Abstract

**Background:**

Evidence regarding blood pressure (BP) variability (BPV) and its independent association with adverse outcomes has grown. Diabetic patients might have increased BPV, but there is still an evidence gap regarding relation between BPV and type 2 diabetes beyond mean values of BP.

**Objective:**

To examine the relationship between 24 h ambulatory BP monitoring (ABPM, short-term variability) and visit-to-visit in-office BPV (OBP, long-term variability), in diabetics (*D*) and nondiabetics (ND), and to explore BPV relation with estimated glomerular filtration rate (eGFR), and pulse wave velocity (PWV) as indicators of target organ lesion.

**Materials and Methods:**

We conducted a single-center cross-sectional study in an outpatient BP unit, including adult patients consecutively admitted from 1999 to 2019. Multivariate was performed to compare BPV between *D* and ND adjusted for clinical variables. Pearson's correlation was performed to evaluate relation of BPV with eGFR and PWV.

**Results:**

A total of 1123 patients with ABPM and OBP measurements were included. Values of eGFR and PWV were worse in *D* than in ND. Measurements of OBPV did not differ between groups. Of ABPM BPV, the coefficient of variation and standard deviation for daytime systolic BP were higher in *D* compared to ND, but only in ND did BPV correlated with both eGFR and PWV.

**Conclusion:**

We found that diabetes is associated with higher variability of daytime BP than nondiabetics along with worse damage of vascular and renal function. However, in contrast to nondiabetics, in diabetics eGFR and PWV may not be dependent on BP variability, suggesting that other mechanisms might explain more rigorously the greater damage of target organ lesion markers.

## 1. Introduction

High blood pressure (BP) is a well-known risk factor for cardiovascular mortality and morbidity, and the main contributor to global disease burden [1]. In addition to the mean of office blood pressure measurements, and with the crosswise use of ABPM in clinical practice, evidence regarding BP variability (BPV) and its independent association with adverse outcomes has grown [2].

BPV can be classified into short-term, mid-term, and long-term variability. Seriated office BP (OBP) over the months and years might be considered as long-term BPV, seriated home BP measurement (HBPM) over a week as mid-term BPV, and (ABPM) as short-term BPV, and all have been related to cardiovascular adverse outcomes [3, 4].

In diabetic patients, both DMT1 and DMT2, atherosclerosis, and microvascular diseases, such as nephropathy, are signs of a poorly managed condition [5, 6]. Hypertension's impact in an organ damage is incremental to diabetes [7], and these patients might have increased BPV through different mechanisms, including increased arterial stiffness and the development of autonomic dysfunction [2]. Evidence regarding relation between BPV and type 2 diabetes beyond mean values of BP remains to be clarified.

Thus, our aim was to examine the relationship of short-term and long-term BPV with diabetes and interaction of target organ lesion indicators (estimated glomerular filtration rate (eGFR) and pulse wave velocity) in this relationship.

## 2. Materials and Methods

We performed a cross-sectional study in the outpatient clinic of Blood Pressure Unit, Hospital Pedro Hispano, Matosinhos, Portugal, an Excellence Center of the European Society of Hypertension [8]. The study was carried in full accordance with the guides of the Declaration of Helsinki, all subjects followed the routine clinical procedures and gave their informed consent, and all data collection was approved by the local Hospital Ethical Committee. Patients included were Caucasian, aged between 18 and 75 years, admitted to the Blood Pressure Unit, Hospital Pedro Hispano, Matosinhos, Portugal from 1999 to 2019.

Patients underwent demographic and clinical baseline data collection either by questionnaire in the first appointment or from clinical files: age, gender, weight and height, family history of cardiovascular risk and adverse outcomes, and calculated body mass index (BMI). Clinical analysis, collected within 3 months from first appointment included glycated hemoglobin (HbA1C), fasting plasma glucose (FPG), and 24 h urinary sodium and potassium, and as indicators of target organ lesion: estimated glomerular function according to MDRD formula (eGFR) and pulse wave velocity (PWV). Patients were excluded if they had a significant inflammatory disease, if they had a change in their ongoing therapy in the last 3 months, or if they were pregnant, critically ill, or had a life expectancy under 3 months. Patients were examined under their stable chronic therapies and habitual dietary and physical activity habits.

Diabetes mellitus was defined by two fasting plasma glucose ≥ 126 mg/dl, 2 h post-load plasma glucose ≥ 200 mg/dL, HbA1C ≥ 6.5%, or use of antidiabetic agents or personal history of diabetes [6, 9]. Pulse wave velocity (PWV), as an indicator of target organ lesion (atherosclerosis), was automatically calculated (as the ratio between distance and transit time) based in two Doppler pulse flow waves recordings simultaneously obtained at the level of the right common carotid and right femoral arteries, as reported previously [8], using a validated noninvasive device (Complior; Colson, Garges les Gonesse, France). PWV was only available for 37.9% of patients. Patients were categorized in four circadian patterns according to nocturnal SBP fall, assessed as the continuous night-to-day ratio (NDR), transformed into percent reduction of daytime values: normal dippers (NDR = ]0.8; 0.9]), extreme dippers(NDR ≤ 0.8), reduced dippers(NDR = ]0.9; 1.0]), and reverse dippers(NDR>1.0).

Seriated OBP and in-office heart rate (measured by arterial peripheral pulse) measurements were collected in 3 consecutive clinical appointments in the unit, within a 6-month interval from each other. ABPM and OBP measurements were taken as reported in our previous work and performed according to the American Heart Association 2018 recommendations [7, 8]. ABPM monitoring was carried out using Spacelabs 90207 and 90217 (Spacelabs, Redmond, Washington, USA), and OBP recordings were measured using automatic sphygmomanometer OMROM models 705-IT and M4-I (Omron Healthcare, Hoofddorp, The Netherlands). ABPM data were divided into daytime and night-time according to patients' reports, to compare these different time-sets of ABPM (mean measurements during daytime, night-time, and 24 h) and consider circadian variations of BP.

BPV was measured by the following parameters: delta systolic/diastolic blood pressure (DS/DBP; calculated as the absolute difference between the maximum and minimum systolic/diastolic BP value, respectively); coefficient of variation (CV; calculated as SD/mean pressure x 100%); standard deviation (SD); average real variability (ARV), computed as the average of the absolute differences between consecutive BP, reflecting reading-to-reading, within-subject variability in BP or pulse levels; and “weighted” 24-hour SD (wSD; computed as the average of day and night SDs, weighted for their respective durations, as reported in Bilo et al. [10]), that can minimize the effect of nocturnal dipping without discarding information about BPV.

Statistical analysis was computed using an IBM SPSS software (version 26; SPSS Inc, Chicago). Most of the continuous variables assumed a non-normal distribution. After visual analysis and the Kolmogorov–Smirnov test, only age, 24 h urinary sodium, daytime/night-time/24H pulse rate, in-office SD DBP, night-time SD DBP, daytime DBP, and eGFR presented a normal distribution (*P* > 0.05); other BPV variables were right-skewed. To compare between nondiabetic (ND) patients and diabetic (*D*) patients a significance level (*α*) of 0.05 was considered and Pearson's chi-square and Mann–Whitney rank sum tests were applied. We then performed generalized linear regression analysis (gamma distribution with log link function, considering maximum likelihood as estimation method) for BPV variables that were significantly correlated with diabetes (Table 1) in univariate analysis, adjusted for significant clinical variables in univariate analysis (Table 2), and respective BP mean. Spearman's correlation coefficients (Rs) were calculated for the relationship between target organ lesion indicators (creatinine clearance and pulse wave velocity) and significant BPV variables after adjustment. Correlations were described as negligible, weak, moderate, strong, and very strong as reported by Prion and Haerling [11].

## 3. Results

### 3.1. Population Characteristics

A total of 1123 patients (851 nondiabetics and 272 diabetics) were included. The demographic characteristics, previous medical history, family history, and clinical analysis are presented in Table 2. In the diabetic group, 52% of patients were male, 61% had dyslipidemia, patients were older, and had higher BMI. Family history of diabetes was significantly higher and family history of hypertension was significantly lower in the *D* group. All variables measured in clinical analysis were significantly different between the groups. In particular, PWV values were higher and eGFR values lower in diabetics than in nondiabetics. All mean systolic blood pressure values (in-office and 24 h-ABPM) were significantly higher in diabetics vs. nondiabetics, but no differences were found for mean diastolic blood pressure values.

### 3.2. Long-Term BPV

As shown in Table 3, values of long-term BP variability (BP in-office) were not significantly different between the groups (Table 3).

### 3.3. Short-Term BPV

Table 1 shows the results of short-term BP variability (24 h-ABPM). Diabetic patients showed higher values of daytime systolic BP variability than nondiabetics and this was the only significant difference on 24 h-ABPM variability indices between these groups.

There was also a differential distribution of circadian type between diabetics and nondiabetics (Pearson chi-square *P*-value 0.003), with the double prevalence of reverse dipper profile in diabetic patients: 37 (14%) of diabetics were reverse dippers vs. 57 (7%) of nondiabetics (Table 2).

Multivariate generalized linear regression analysis for each BPV variable that significantly related to diabetes in univariate analysis was performed to evaluate association with diabetes. Adjustments were performed for age, BMI, dyslipidemia, familiar history of diabetes, and hypertension; a respective BP value was also included in the model (for ARVS and wSD, SBP 24 h was considered). After adjustment, only 24-hours delta SBP and daytime systolic BPV variables (SD and CVS) were independently correlated with diabetes (*P*=0.042, *P*=0.040) (Table 1).

### 3.4. Correlation with Target Organ Lesion Indicators

In the overall population, significant correlations (*P* < 0.001) were observed between age and both PWV (Rs for PWV = 0.51) and eGFR (Rs = − 0.49). PWV and eGFR were negatively correlated with each other (Rs = − 0.29, *P* < 0.001). Daytime systolic SD and CVS were significantly correlated with both PWV (Rs = 0.39 and 0.26, respectively, *P* < 0.001) and eGFR (Rs = − 0.19 and − 0.18, respectively, *P* < 0.001).

### 3.5. Age

**Figure 1:**
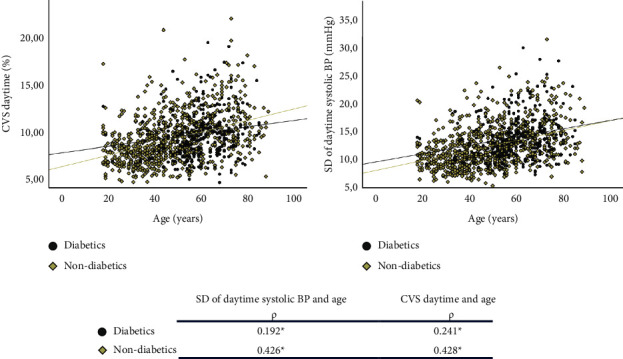
Evaluation of interaction of diabetes and age.^*∗*^*P* < 0.05, the Pearson's correlation test, SD: standard deviation, CVS: coefficient of variation of systolic blood pressure

Age was significantly correlated with all systolic BPV variables, with moderate correlations for daytime BPV, weak for 24 h BPV and negligible for night-time BPV. In Figure 1, daytime systolic SD and CVS were plotted for evaluation of interaction of diabetes and age. Age remained significantly correlated to daytime systolic SD and CVS, with moderate correlation in nondiabetics and negligibly to weakly correlated in diabetic patients, with significant differences between diabetics and nondiabetic slopes for the correlation between age and daytime systolic SD (*P*=0.04).

### 3.6. Short-Term BPV

**Figure 2:**
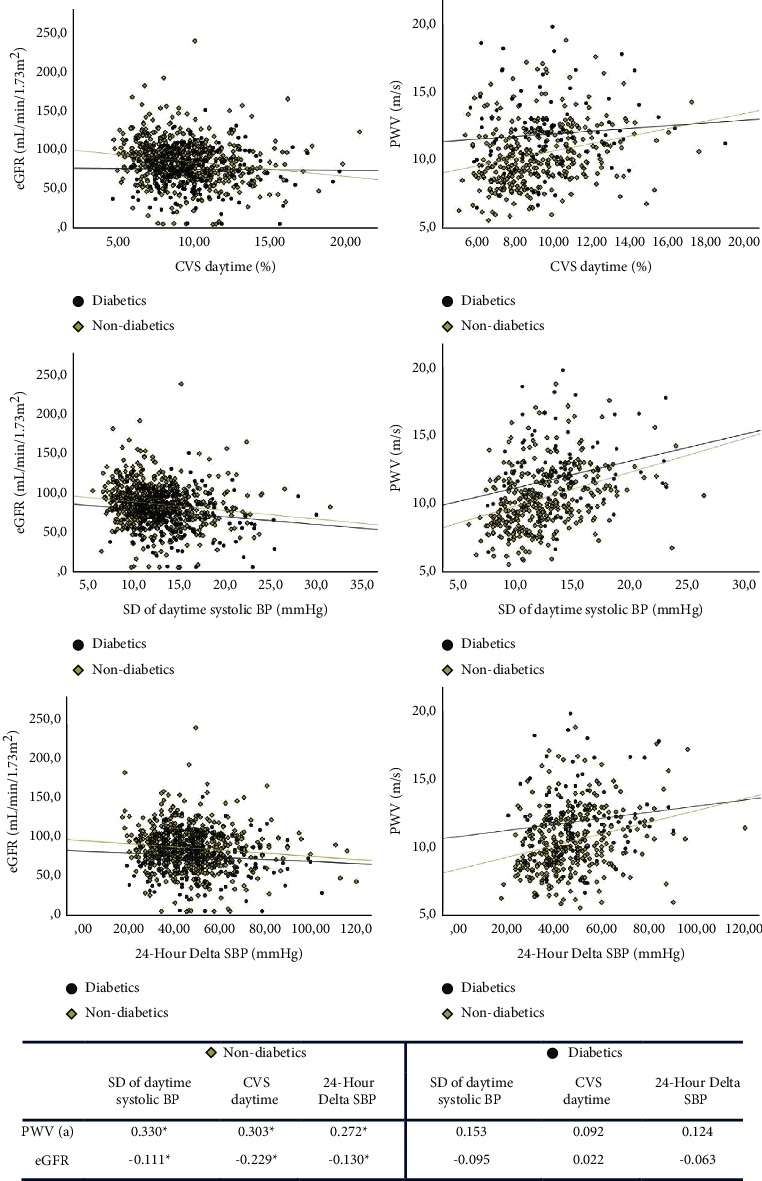
Relationship between BPV and indicators of target organ lesion. ^*∗*^*P* < 0.001; (a) data only available for 37.9% of patients with non-differential missing data between diabetics and nondiabetics, the Pearson's correlation test, DSBP: delta (maximum-minimum) systolic blood pressure, SD: standard deviation, CVS: coefficient of variation of systolic blood pressure.

The relationship between daytime systolic SD and CVS with target organ lesion indicators (estimated a glomerular filtration rate and pulse wave velocity), by groups are plotted in Figure 2. As shown in Figure 2, in nondiabetics, daytime systolic BP variability correlated significantly positively with PWV and negatively with eGFR values, but no such correlations were ever found in diabetic patients.

## 4. Discussion

In the present study, we assessed BP variability in nondiabetics and diabetic patients with 24 h-ABPM and seriated OBP (from 3 consecutive outpatient evaluations). We applied different formulas of assessment of variability to evaluate the association of BPV with diabetes and, also, the association of BPV with vascular, renal, and target organ lesion indicators (PWV and eGFR, respectively). Our population of diabetics was older, weighted more, and had worse clinical conditions than nondiabetics as expressed by higher PWV and lower eGFR. Although no differences in long-term BP variability were observed between groups, variability of daytime systolic BP was higher in diabetics than nondiabetics, even after adjustment for other significant clinical variables, as expected [9]; in univariate analysis, diabetic patients had higher values of systolic BP variability, and after adjustment for age, BMI, dyslipidemia, familiar history of diabetes and hypertension and a respective BP value, only daytime systolic SD and CVS remained significantly higher in diabetics. These results are in agreement with those of Casali et al. [4].

### 4.1. Long-Term BPV Differences between Diabetics and Nondiabetics

Previous evidence suggested that increasing values of long-term BPV predict the development and progression of diabetes organ target lesions, such as nephropathy, including correlation with PWV and urinary albumin excretion, another indicator of kidney lesion [1, 12]. Yet, the mechanism to explain its contribution as a predictor is still debatable, with a relevant contribution of behavioral influences and even seasonal related climatic changes [1]. In our study, we did not find significant differences between long-term BPV in diabetics vs. nondiabetics, suggesting that long-term BPV does not contribute to the different odds of cardiovascular complications in diabetic vs. nondiabetic hypertensive patients.

### 4.2. Short-Term BPV Differences between Diabetics and Nondiabetics

Regarding short-term BPV (in 24 h-ABPM): at the moment, there is no standard way of measuring BPV, being one of the strengths of our study the use of different parameters to measure it, allowing for a more comprehensive evaluation of BPV and comparison with further research [3].

The prevalence of reverse dippers was significantly higher in diabetics vs. nondiabetics. This circadian pattern has been related to increased cardiovascular risk, and this association with diabetes has been already explored before [13].

Independently of the measurement, systolic BPV has been increasingly associated with cardiovascular outcomes in diabetic and nondiabetic patients, an impact that might go beyond that of BP [3, 14]. Chiriacò et al. suggested that it could be a relevant factor to include in adverse outcome risk prediction for diabetic patients [3], taking into account measurement limitations and each measurement association with the diabetes itself. As proposed by Parati et al., long-term BPV and short-term BPV probably translate the action of different physiological mechanisms [8]. Considering that the latter were significantly different between diabetics and nondiabetics, it may more accurately reflect the impact of diabetic aggression, through either autonomic modulation or atherosclerosis enhanced augmentation [1, 15].

### 4.3. Correlation of Short-Term BPV and Target Organ Lesion

In our study, short-term BPV was higher in diabetics than in nondiabetics. However, short-term BPV was indeed significantly correlated, in nondiabetics, with increased PWV and lower eGFR, but in diabetics there was no correlation of these markers of target organ lesions and BPV. Looking closer at the results of Chiriacò et al. significant heterogeneity across studies was present, and adverse outcomes were composite measures rather than specific assessments, such as PWV and eGFR, as an estimation of creatinine clearance [3]. Thus, in our study, although BP variability was higher in diabetic patients, its relationship with target organ damage was only observed in nondiabetic patients. These results lead us to speculate that, as proposed by Bell and colleagues, although the artery stiffness and deterioration of renal function in diabetics are worse than in nondiabetics, BPV may not bring additional clinical usefulness to the routinely measured predictors, such as mean blood pressure in diabetics [16].

BPV may indeed be a marker of vascular aging, which may be accelerated in diabetics, but other factors may contribute in a larger scale to it adverse outcomes [16]. In our view, different from nondiabetics, the diabetic condition may cause structural renal and vascular damages by specific diabetic abnormalities independently of BP variability and likely of other cardiovascular risk factors. In support of this conjecture, we found that the variable age was significantly correlated with all systolic BPV variables, but its correlation with systolic BPV was significantly higher in nondiabetic vs. diabetics, with correlation becoming negligibly to weakly correlate in diabetic patients. It is well established that cardiovascular risk is naturally higher in diabetics than nondiabetics, which is confirmed in our study. However, such a higher risk may be related to complex structural and metabolic abnormalities, such as inflammation, endothelial dysfunction, oxidative stress, fibrosis, accumulation of AGEs, and atherosclerosis. [17–19], most of them escape from the dependence of BP variability. In other words, our data suggest that diabetic patients exhibit greater BP variability, more severe organ damage but less dependence on BP variability and probably of other usual anthropometric variables like age, gender, etc.

### 4.4. Strengths and Limitations

This cross-sectional study's strengths include a large cohort of patients with and without type 2 diabetes. The comparison between diabetics and nondiabetics allowed for a distinction of the BPV impact beyond diabetes. Although subgroup analysis of patients on BP lowering drugs within diabetic population has already been reported [16], most of the existing literature focused only on overall diabetic population and [12, 15], as our results present, there seems to be a relevant differential association of BPV to PWV and eGFR in nondiabetics vs. diabetics. As mentioned above, we have also computed several different parameters to measure BPV, exploring simple and more complex measurements enhancing the comparability with further literature.

The main limitation of our study is its observational cross-sectional nature and the fact it was conducted in a single center. Although we have considered several potential confounders, other characteristics, such as diabetes duration disease, anti-hypertensive drugs, and diabetes treatment could be considered. We have performed complete-case analysis, considering that missing data were not differential between diabetics and nondiabetics. PWV was only available for 37.9% of patients. Considering this is a cross-sectional study, dealing with observational data collected from patients' clinical registries, missing data imputations was not performed [20]. For GLM, SPSS automatically excludes cases with system-missing values for any of the variables on the GLM variable list, and user-missing values were treated as valid (data from 146 patients were excluded from GLM of 13%). As stated by STROBE, this alternative for missing data management may still be biased [20], therefore, further large-scale studies are needed to explore relation of BPV and PWV.

## 5. Conclusion

In conclusion, we found that diabetes is associated with higher variability of daytime BP than nondiabetics, along with worse damage of vascular and renal function. However, in contrast to nondiabetics, in diabetics eGFR and PWV may be not dependent of BP variability, suggesting that other mechanisms might explain more rigorously the greater damage of target organ lesion markers.

## Figures and Tables

**Table 1:** Short-term BP variability SD and CV comparison between ND and D across 24 h, daytime, and night-time.

	Non-diabetics	Diabetics	*P* value between groups	Adjusted *P* value
Delta (mmHg)				
DSBP 24 h	46.0 (37.0–55.0)	48.0 (38.0–59.0)	^*∗*^=0.006	^*∗*^0.006
DDBP 24 h	36.0 (30.0–42.0)	33.0 (27.0–40.0)	^*∗*^=0.001	0.758
SD systolic (mmHg)				
24 h	13.6 (11.4–16.3)	14.4 (12.6–17.6)	^*∗*^<0.001	0.104
Daytime	11.9 (9.9–14.4)	13.6 (11.1–16.1)	^*∗*^<0.001	^*∗*^0.042
Night-time	10.0 (7.9–12.4)	11.1 (8.7–13.7)	^*∗*^<0.001	0.112
SD diastolic (mmHg)				
24 h	10.6 (9.1–12.5)	10.2 (8.6–12.1)	^*∗*^0.010	0.687
Daytime	8.9 (7.6–10.5)	8.8 (7.4–10.4)	=0.661	—
Night-time	8.1 (6.4–10.1)	8.4 (6.6–10.4)	=0.598	—
CVS (%)				
24 h	10.6 (8.9–12.4)	10.5 (9.1–12.5)	=0.410	—
Daytime	8.8 (7.4–10.7)	9.5 (8.0–10.9)	^*∗*^0.001	^*∗*^0.040
Night-time	8.4 (6.6–10.5)	8.6 (6.6–10.7)	=0.424	—
CVD (%)				
24 h	13.7 (11.6–16.1)	13.3 (11.2–15.4)	^*∗*^0.021	0.967
Daytime	11.0 (9.2–13.1)	10.9 (9.3–12.8)	=0.962	—
Night-time	11.7 (9.2–14.6)	11.9 (9.3–14.4)	=0.900	—
ARVS	8.4 (6.9–10.4)	9.3 (7.7–12.0)	<0.001	0.211
ARVD	6.5 (5.5–7.8)	6.5 (5.3–8.0)	=0.541	—
wSD systolic 24 h	8.6 (7.1–10.2)	9.3 (7.7–12.0)	<0.001	0.179

DSBP: delta (maximum-minimum) systolic blood pressure, DDBP: delta diastolic blood pressure, SD: standard deviation, CVS: coefficient of variation of systolic blood pressure, CVD: coefficient of variation of diastolic blood pressure, CVP: coefficient of variation of arterial peripheral pulse, ARVS/D/P: average real variability of systolic/diastolic/arterial peripheral pulse ND: nondiabetics, D: diabetics. Variables are presented as medians (interquartile range: percentile 25-percentile 75) and comparisons between ND vs. D were tested with the Mann–Whitney rank sum test. A GLM model was computed to adjust for age, BMI, dyslipidemia, familiar history of diabetes and hypertension, and mean BP value.

**Table 2:** Clinical characteristics of nondiabetics and diabetics.

	Nondiabetics	Diabetics	Total	*P* value
N(%)	851 (76)	272 (24)	1123	
Age (years)	48 (36–61)	60 (53–68)	53 (39–64)	<0.001
Male	346 (41)	141 (52)	487 (43)	0.001
BMI (Kg/m2)	27.4 (24.5–31.0)	29.1 (26.0–32.5)	27.9 (24.7–31.4)	<0.001
Smokers	124 (15)	37 (14)	161 (14)	0.692
Dyslipidemia	298 (35)	165 (61)	463 (41)	<0.001
Family history N(%)				
Hypertension	293 (34)	69 (25)	362 (32)	0.005
Stroke	57 (7)	11 (4)	68 (6)	0.110
Coronary artery disease	90 (11)	25 (9)	115 (10)	0.512
Diabetes	122 (14)	62 (23)	184 (16)	0.001
Clinical analysis				
Fasting glucose (mg/dL)	93 (85–101)	135 (119–167)	98 (87–114)	<0.001
HbA1C (%)	5.6 (5.3–5.8)	6.8 (6.0–7.7)	5.9 (5.4–6.7)	<0.001
Creatinine (mg/dL)	0.80 (0.70–1.00)	0.90 (0.80–1.10)	0.80 (0.70–1.00)	<0.001
eGFR (mL/min/1.73 m2)	86.6 (70.7–100.7)	75.3 (60.0–94.4)	84.2 (67.6–98.9)	<0.001
24 h Urinary sodium (mEq/24 h)	183 (140–244)	201 (158–269)	189 (142–251)	0.015
24 h Urinary potassium (mEq/24 h)	68 (53–85)	91 (70–109)	73 (57–95)	<0.001
PWV (m/s) (a)	10.1 (8.8–12.0)	11.8 (10.0–13.0)	10.5 (9.0–12.2)	<0.001
BP analysis				
OBP systolic/diastolic (mmHg)	147 (136–160)/92 (83–101)	160 (145–178)/90 (81–99)	150 (137–165)/92 (82–100)	<0.001/0.108
24 h-ABPM SBP/DBP (mmHg)	129 (121–138)/78 (71–85)	139 (129–150)/78 (70–85)	131 (122–141)/78 (71–85)	<0.001/0.386
Daytime SBP/DBP (mmHg)	133 (125–143)/82 (74–90)	143 (132–155)/81 (74–88)	135 (126–146)/82 (74–89)	<0.001/0.107
Nighttime SBP/DBP (mmHg)	118 (110–128)/69 (62–76)	129 (118–142)/70 (63–77)	120 (111–131)/69 (63–76)	<0.001/0.154
Circadian profile (b)				
Dipper	368	101	469	
Non-dipper	346	111	457	0.003
Reverse dipper	57	37	94	
Extreme dipper	73	20	93	

BMI: body mass index, HbA1c: glycated hemoglobin, eGFR: estimated glomerular filtration rate, PWV: pulse wave velocity, OBP: in-office blood pressure (mean of 3 measurements at baseline), 24 h-ABPM: 24hambulatory blood pressure monitoring, SBP: systolic blood pressure, DBP: diastolic blood pressure. Continuous variables are presented as medians (interquartile range: percentile 25-percentile 75) and categorical variables are presented as absolute frequency (%). Comparisons were tested with the Mann–Whitney rank sum test and the X2 test. (a) data only available for 37.9% of patients with non-differential missing data between diabetics and nondiabetics, (b) missing data for 10 patients.

**Table 3:** Long-term (in-office) BP variability.

	Nondiabetics	Diabetics	Total	*P* Value between groups
DSBP (mmHg)	16 (8–27)	17 (10–29)	16 (9–28)	0.295
DDBP (mmHg)	11 (6–18)	12 (6–17)	11 (6–17)	0.803
SD systolic (mmHg)	12.8 (7.8–19.8)	13.0 (8.3–21.0)	12.8 (8.0–20.1)	0.074
SD diastolic (mmHg)	7.8 (5.2–11.4)	8.5 (5.5–11.7)	8.0 (5.3–11.6)	0.318
SD pulse (bpm)	7.8 (4.4–12.1)	6.4 (3.8–11.3)	7.6 (4.2–12.0)	0.055
CVS (%)	8.7 (5.3–13.4)	8.75 (5.72–13.46)	8.8 (5.4–13.4)	0.806
CVD (%)	9.2 (5.9–13.1)	9.86 (6.39–13.65)	9.3 (6.0–13.3)	0.141
CVP (%)	10.14 (5.9–15.7)	9.0 (5.1–14.4)	9.9 (5.7–15.3)	0.050
ARVS	6.0 (3.0–11.3)	6.3 (3.3–13.0)	6.3 (3.0–11.7)	0.118
ARVD	3.7 (1.7–6.2)	3.3 (1.5–6.0)	3.5 (1.7–6.0)	0.632
ARVP	5.0 (2.5–9.5)	4.5 (2.0–9.0)	5.0 (2.5–9.0)	0.322

SD: standard deviation, DSBP: delta (maximum-minimum) systolic blood pressure, DDBP: delta diastolic blood pressure, SD: standard deviation, CVS/D/P: coefficient of variation of systolic blood pressure/diastolic blood pressure/arterial peripheral pulse, ARVS/D/P: average real variability of systolic/diastolic/arterial peripheral pulse, ND: nondiabetics, D: diabetics. Variables are presented as medians (interquartile range: percentile 25-percentile 75) and comparisons between ND vs. *D* were tested with the Mann–Whitney rank sum test.

## Data Availability

Data supporting the results can be requested directly from the corresponding author.
